# Spraying alginate oligosaccharide improves photosynthetic performance and sugar accumulation in citrus by regulating antioxidant system and related gene expression

**DOI:** 10.3389/fpls.2022.1108848

**Published:** 2023-01-30

**Authors:** Zhiming Li, Songpo Duan, Bosi Lu, Chunmei Yang, Hanqing Ding, Hong Shen

**Affiliations:** ^1^ College of Natural Resources and Environment, South China Agricultural University, Guangzhou, China; ^2^ Guangdong Nongken Tropical Agriculture Research Institute Co., Guangzhou, China

**Keywords:** biostimulants, seaweed extracts, antioxidative enzyme, assimilate, sugar metabolism

## Abstract

Alginate oligosaccharides (AOS) are functional substances in seaweed extracts that regulate crop quality and stress tolerance. In this paper, the effects of AOS spray application on the antioxidant system, photosynthesis and fruit sugar accumulation in citrus was investigated through a two-year field experiment. The results showed that 8-10 spray cycles of 300-500 mg L^-1^ AOS (once per 15 days) increased soluble sugar and soluble solid contents by 7.74-15.79% and 9.98-15.35%, respectively, from citrus fruit expansion to harvesting. Compared with the control, the antioxidant enzyme activity and the expression of some related genes in citrus leaves started to increase significantly after the 1st AOS spray application, while the net photosynthetic rate of leaves increased obviously only after the 3rd AOS spray cycle, and the soluble sugar content of AOS-treated leaves increased by 8.43-12.96% at harvest. This suggests that AOS may enhance photosynthesis and sugar accumulation in leaves by antioxidant system regulation. Moreover, analysis of fruit sugar metabolism showed that during the 3rd to 8th AOS spray cycles, AOS treatment increased the activity of enzymes related to sucrose synthesis (SPS, SSs), upregulated the expression of sucrose metabolism (*CitSPS1*, *CitSPS2*, *SUS*) and transport (*SUC3*, *SUC4*) genes, and promoted the accumulation of sucrose, glucose and fructose in fruits. Notably, the concentration of soluble sugars in citrus fruits was significantly reduced at all treatments with 40% reduction in leaves of the same branch, but the loss of soluble sugars in AOS-treated fruits (18.18%) was higher than that in the control treatment (14.10%). It showed that there was a positive effect of AOS application on leaf assimilation product transport and fruit sugar accumulation. In summary, AOS application may improve fruit sugar accumulation and quality by regulating the leaf antioxidant system, increasing the photosynthetic rate and assimilate product accumulation, and promoting sugar transfer from leaves to fruits. This study shows the potential application of AOS in the production of citrus fruits for sugar enhancement.

## 1 Introduction

Citrus are one of the most important sources of natural nutrient supplements for the human body, providing a wide range of nutrients, and they are also the most widely grown fruit crops in China, with an annual production of more than 45 million tons and great industrial value (Sheng et al., 2017; Batista-Silva et al., 2018). However, inconsistent fruit quality has been a bottleneck limiting the development of the citrus industry. The soluble sugar content in citrus fruits is an important indicator of their quality, directly affecting consumer preference and purchase choices (Lin et al., 2015; Guo et al., 2016). However, sugar accumulation in fruits is a complex process that is strongly influenced by multiple factors, such as highly regulated carbohydrate transport and metabolism as well as the external environment. Considering the contradiction between long breeding cycles and urgent market demand, rapid and effective improvement of sugar accumulation and fruit quality is a top priority for researchers. Many studies have shown that the use of exogenous harmless natural biostimulants, including natural biostimulants, plant growth regulators and antioxidants, may be a good strategy to improve fruit quality (Cai et al., 2014; Raza et al., 2014; Zhao et al., 2020).

Alginate oligosaccharides (AOS) are low-molecular-weight polymers generated by the degradation of alginate and consist of 2-20 monosaccharides (Courtois, 2009). These biostimulants have the advantages of low molecular weight, high solubility and stability, easy absorption by the body and natural nontoxicity and exhibit great potential for application (Liu et al., 2019a). The available literature reports that AOS can act as signaling molecules or excitons when they enter plants, participating in a variety of physiological activities in plants and regulating plant growth and development. For example, AOS can activate the accumulation of *β*-amylase in maize, promote root growth in lettuce, mustard, barley and soybean seedlings, and increase the levels of free proline, total soluble solids and abscisic acid in tomato (Keith, 2003; Liu et al., 2009; Aftab et al., 2011; Luan et al., 2012). AOS can also reduce oxidative damage and maintain metabolic balance in crops by activating antioxidant enzymes such as catalase (CAT), superoxide dismutase (SOD), and peroxidase (POD), which in turn enhance crop resistance to stressful environments (Zhang et al., 2020). Therefore, AOS application at the right time, at the right dose, or in the right form can enable the realization of their potential benefits in plant.

Sugar accumulation in citrus is influenced by leaf photosynthesis and multiple sucrose-metabolizing enzymes in the fruit. During fruit development, sucrose in fruit is degraded by acid convertase (AI), neutral convertase (NI) and sucrose synthase-catabolic direction (SSc) to form fructose and glucose, which is then resynthesized to sucrose by sucrose phosphate synthase (SPS) and sucrose synthase-synthetic direction (SSs) phosphorylation (Shen et al., 2017). The photosynthetic products of leaves (source) are the main source of sucrose accumulation in citrus fruits (reservoir). Chloroplasts in leaf pulp cells convert carbon dioxide (CO_2_) to propyl phosphate *via* the tricarboxylic acid cycle under light conditions, and propyl phosphate is transferred from chloroplasts to cytoplasm *via* transporter to further synthesize propyl 6-phosphate. While propanose 6-phosphate and UDP-glucose (UDPG) are synthesized into sucrose under the catalytic conditions of sucrose phosphate synthase (SPS) and sucrose phosphorylase (SPP) and then transported to the fruit (Fu et al., 2011; Chen et al., 2012; Ruan, 2014). However, maintaining good photosynthesis requires that the leaves remain in a favorable environment, which is a challenge in citrus production, as during citrus development, the plants are often under biotic or abiotic stress of varying intensity (Sina et al., 2021). Previous studies have shown that the application of algal oligosaccharides can improve the light and action capacity and activate the antioxidant system in crops under conditions such as drought, salinity, extreme weather or disease (Liu et al., 2009; Kailemia et al., 2014; Zhang et al., 2020). Rice seedlings also produced more CAT, POD, and SOD enzymes and exhibited improved leaf photosynthesis when AOS with an average polymerization index of 13.9 at 3000 μg mL^-1^ were applied through spraying (Qiao and Ouyang, 2013). AOS application promotes crop photosynthesis and metabolite accumulation by alleviating the environmental stress on the crop through activation of the antioxidant system to alleviate unfavorable survival conditions. In addition, AOS can accelerate growth hormone biosynthesis and transport in crops by upregulating the expression of growth hormone-related genes such as *OsYUCCA1*, *OsYUCCA5*, *OsIAA11*, and *OsPIN1* (Zhang et al., 2014; Zhang et al., 2015). Therefore, spray application of AOS may be beneficial to fruit growth and sugar accumulation.

AOS have been shown to have positive effects on crops in terms of yield promotion and induction of resistance. However, studies on the effect of AOS in enhancing sugar accumulation and improving the quality of citrus fruits, as well as the underlying mechanisms of action, have not been reported. Therefore, the following hypotheses are proposed: (1) AOS spray application could increase fruit sugar accumulation and improve citrus quality, and (2) AOS affect sugar accumulation by regulating the leaf antioxidant system, increasing the photosynthetic rate, and promoting sugar transfer from leaves to fruits. To verify this correlation, the effects of AOS spray application on the citrus leaf antioxidant system, photosynthesis and fruit sugar accumulation were investigated through a two-year field experiment (2020-2021), and the mechanism underlying the effect of AOS action on citrus fruit sugar metabolism was preliminarily investigated. This study provides new insights into the enhancement of sugar production in citrus fruits.

## 2 Materials and methods

### 2.1 Preparation and characterization of AOS

Referring to our previous study (Yang et al., 2020). Briefly, a 5% (w/v) solution of sodium alginate was prepared with deionized water, and the alginate lyase AlgSH7 (produced by the algal-degrading strain *Microbacterium* sp. SH-1) was added and allowed to react at 40°C for 24 h. Then, the undegraded product was precipitated with 4 times the volume of ethanol and centrifuged, and the supernatant was freeze-dried to prepare the AOS. The products were qualitatively and quantitatively analyzed by thin-layer chromatography (TLC), electrospray ionization–mass spectrometry (ESI–MS, Bruker, Germany) and high-performance liquid chromatography (HPLC, Agilent 1200, United States). The results are shown in **Supplementary Figure S1**. The main components of this AOS product are oligosaccharide complexes with degrees of polymerization(DP) 2, 3, 4 and 5, percentages of each component are 11.39, 19.25, 40.81 and 23.51%, respectively.

### 2.2 Experimental site and experimental design

#### 2.2.1 Field experiment

**Figure 1:**
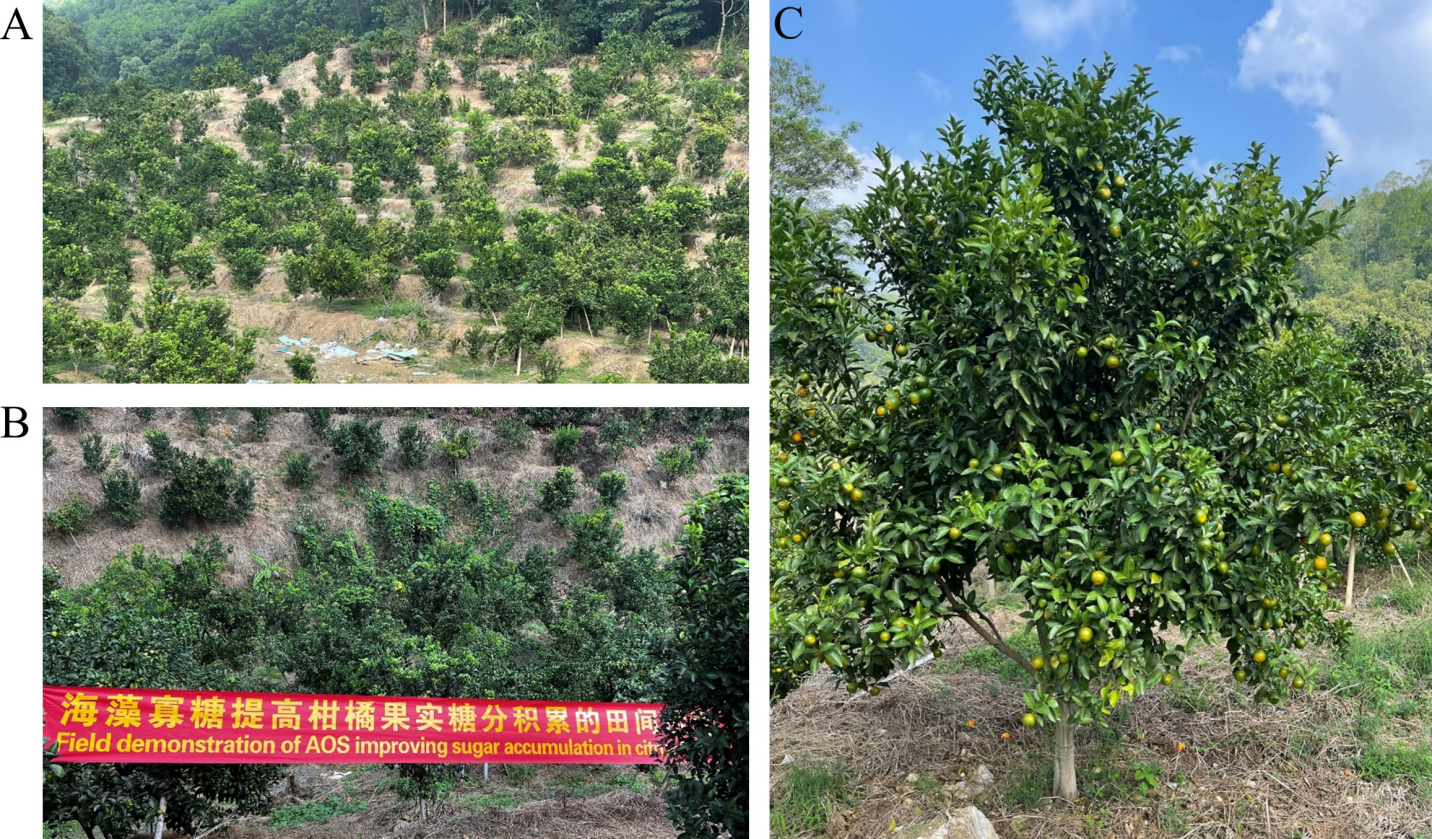
Citrus test site scenario map **(A, B)**, Citrus fruit tree diagram **(C)**.

The experimental orchard was located in Shuikoukan village, Zhengguo town, Zengcheng District, Guangzhou city, Guangdong Province, China (N 23°27′36.85″, E 113°49′28.48″). The soil properties were as follows: organic matter, 21.90 g kg^-1^; alkaline decomposed nitrogen, 138.29 mg kg^-1^; effective phosphorus, 38.83 mg kg^-1^; effective potassium, 170.32 mg kg^-1^; and pH, 6.10. The test citrus trees were 5–7 year–old “Shatangju” fruit trees. The experiment was set up with three levels of AOS spray application: 0 mg L^-1^, 300 mg L^-1^ and 500 mg L^-1^, recorded as CK, 300AOS and 500AOS, respectively. There were 4 replications and 12 citrus trees in each treatment. Spray application in 2020 began in mid-July and was performed, once every 15 days, for a total of 10 spray cycles. Spray application in 2021 began in early August and was performed, once every 15 days, for a total of 8 spray cycles. Each tree was treated with 2 L of solution per spray. Leaves and fruits of each treatment were collected for assimilation product and quality determination during the harvesting period at the orchard (December 10, 2020, and December 5, 2021). In addition, in the 2021 experiment, leaves and fruits were collected at 30-d intervals starting from the spray application treatments for a total of four times (recorded as 1 time, 3 times, 5 times and 8 times, respectively). These samples were used for the detection of leaf antioxidant enzyme activities, photosynthesis and fruit sugar fractions, and sample collection and photosynthesis were performed on the morning of the second day after each AOS spray application. Specific orchard scenarios, treatments and collection times are shown in **Figure 1** and **Supplementary Table S1**. Fruit samples were collected as 24 randomly collected representative fruits per treatment at each time point, and leaf samples were collected as functional leaves from around the fruit for each planned collection. There were three periods of fertilizer management during fruit tree planting: (1) 5.0 kg/tree basal fertilizer (1.5 kg of organic fertilizer + 0.2 kg of compound fertilizer) was applied after the harvest of the previous citrus season. (2) 0.5 kg/tree compound fertilizer was applied in mid-April, after the fruit trees had flowered. And (3) 1.0 kg/tree high-potassium compound fertilizer was applied in mid-August. The rest of the routine management, such as dosing, pruning, and insect repellent use, was carried out according to local farming practices, and the rainfall and temperature conditions are shown in **Supplementary Figure S2**.

#### 2.2.2 Leaf reduction experiment

The treatments were set up in 2021 in the above orchard with two levels of AOS application (0 mg L^-1^ and 300 mg L^-1^) and two levels of leaf reduction (no leaf reduction and 40% leaf reduction, recorded as 0%LR and 40%LR, respectively), and the leaf reduction treatments were carried out on two branches of the same citrus tree selected for comparable fruit volume. The reduced leaves were the functional leaves around the fruit. There were 4 replicates of each treatment applied to 12 citrus trees, and the number and timing of the sprays were the same as in the 2021 field experiment. Citrus fruits of each treatment were collected at harvest time and used to test the soluble sugar content and soluble sugar loss rate of the fruits.

### 2.3 Determination of fruit flavor quality

The soluble solid content was determined using a digital glucose meter (TD45, Guangzhou, China). The content of vitamin C (Vc) was determined using molybdenum blue colorimetry (Laurentin and Edwards, 2003). The homogenized juice was titrated with 0.1 mol L^-1^ NaOH to determine the concentration of titratable acid (TA) (Wu et al., 2020). The contents of total soluble sugar and cellulose were determined by using the phenol-concentrated sulfuric acid method and anthrone colorimetry, respectively (Gao, 2006). The sugar-acid ratio was calculated as the ratio of total soluble sugar to titratable acidity.

### 2.4 Analysis of leaf-related indicators

Photosynthesis was measured using a Li–6800 portable photosynthesis measurement system (Li–6800, LI–COR, United States) from 9:00 a.m. to 11:00 a.m. The leaves were selected from the functional leaves around the fruit, and the environmental conditions were as follow: an air flow rate of 500 mL min^-1^, a light quantum flux of 10,000 µmol m^-2^ s (red and blue light sources), a CO_2_ concentration of 410 ± 20 µmol mol^-1^, and an ambient temperature of 35 ± 5°C. The instantaneous net photosynthetic rate (Pn), transpiration rate (Tr), stomatal conductance (Gs) and intercellular CO_2_ concentration (Ci) were measured five times for each citrus tree, and the five measurements was taken (Zhao et al., 2014). The chlorophyll content was determined by selecting leaves around the fruit in the frozen sampling phase, cutting 0.5 g of leaves without veins into 1 mm^2^ pieces, placing them in a 25 mL volumetric flask, bring the volume to 25 ml using 80% acetone (v/v), and extracting the samples in the dark for 24 h until the leaves turned completely white. Absorbance was measured at 664 and 647 nm using a UV-1600 spectrophotometer (UV-1600, Shimadzu, Japan), and the contents of chlorophyll a (Chl a), chlorophyll b (Chl b), and total chlorophyll and the chlorophyll a/b rate were calculated (Ding et al., 2018). The determination of the soluble protein content of leaves was performed by the bicinchoninic acid (BCA) method by extracting leaf tissue with 0.05 M phosphate buffer solution (PBS), incubating the supernatant with BCA reagent at 37°C for 30 min, cooling the solution to room temperature and reading the absorbance of the solution at 562 nm (Smith et al., 1985). The soluble sugar content in the leaves was determined by the sulfuric acid-anthrone method, where in the leaf tissue was extracted with 80% ethanol, the extract was incubated with sulfuric acid-anthrone reagent at 90°C for 15 min, and the absorbance of the solution was measured at 620 nm (Giannakoula et al., 2010). The starch content was measured by the I_2_-KI color development method by homogenizing three 6 mm diameter leaf discs in 2 mL centrifuge tubes with steel beads, heating them in boiling water for 10 min, centrifuging them at 2,500 rpm for 2 min, adding 50 µL of I_2_-KI to the supernatant to colorize the starch, and measuring the absorbance value at a wavelength of 594 nm (Whitaker et al., 2014).

### 2.5 Sugar component extraction and HPLC analysis

Extraction of soluble sugar components: Citrus pulp (2.0 g) was homogenized in 5 mL of 90% ethanol and centrifuged at 10,000 ×g for 15 min at 4°C to collect the supernatant. This procedure was repeated three times, and the collected supernatant was evaporated in an 80°C water bath and then diluted to 10 mL with ultrapure water. After the solution was filtered through a 0.45 μm injection filter, HPLC (Agilent 1200, United States) was used to determine the concentration of soluble sugars (fructose, glucose, and sucrose). To determine the concentrations of sucrose, fructose and glucose, a differential detector and ZORBAX NH2 column (250 mm× 4.6 mm, 5 µm) were used. The chromatographic conditions were as follows: mobile phase, acetonitrile/water (75:25); flow rate, 0.75 mL min^-1^; and column temperature, 25°C.

### 2.6 Analysis of enzyme activities related to leaf antioxidants

Antioxidant enzymes activity were determined using the method of Liu et al. (Liu et al., 2018). Extraction of crude enzyme solution was conducted as follows: 0.5 g of leaves was placed in 5 mL of 50 mmol L^-1^ phosphate buffer (pH 7.8, containing 1% polyvinyl pyrrolidone (PVP)). The enzyme solution was extracted by grinding in an ice bath, homogenized and centrifuged at 12,000 × g and 4°C for 20 min, and the supernatant was taken as the crude enzyme extract.

The SOD activity was assayed by measuring its ability to inhibit the photochemical reduction of nitroblue tetrazolium, with 50% inhibition of the photochemical reduction of NBT as one unit of enzyme activity. The CAT activity was measured as the decline in absorbance at 240 nm due to the decrease of extinction of H_2_O_2_, and a decrease of 0.01 per minute was defined as one unit of enzyme activity. The POD activity was measured as the increase in absor-bance at 470 nm due to guaiacol oxidation, defining an increase in OD of 0.01 per minute as 1 unit of enzyme activity. The APX activity was measured by the decrease in absorbance at 290 nm as ASA was oxidized, measured as the decrease in ASA per minute (Gong et al., 2014).

### 2.7 Analysis of enzyme activities related to sugar metabolism

To extract and determine the AI and NI enzyme activities, 1.0 g of citrus pulp was ground in liquid nitrogen, and then the powder was homogenized with 8 mL of Tris-HCl extraction buffer [pH 7.0, containing 5 mmol L^-1^ MgCl_2_, 2 mmol L^-1^ EDTA-Na_2_, 2% ethylene glycol, 0.2% bovine serum albumin (BSA), 2% PVP, and 5 mmol L^-1^ DL-dithiothreitol (DTT)] and centrifuged at 10,000 ×*g* for 20 min at 2°C. The supernatant was collected, placed in a dialysis bag, and dialyzed overnight with a dialysate (diluted 10-fold with the extraction buffer), and the dialysate was changed twice during the process. After dialysis, the enzyme solution was stored at 4°C to obtain the enzyme extract. The AI enzyme activity assay was carried out in a mixture containing 80 mM sodium citrate buffer (pH 4.5), 100 mM sucrose and enzyme extract. After incubation of this system for 30 min at 37°C, the reaction was terminated by using 0.03 M 3,5–dinitrosalicylic acid (DNS) at 100°C for 5 min. After cooling, the absorbance at 540 nm was recorded. The conditions for the determination of NI activity were similar to those for AI activity except that 100 mM sodium phosphate buffer (pH 7.5) was used. The units of AI and NI are expressed as the amount of glucose produced every hour at pH 4.5 and pH 7.5, respectively (Liu et al., 2019b).

Extraction and determination of SPS, SSs, and SSc enzyme activities were conducted as follows: 1.0 g of citrus pulp was ground in liquid nitrogen and then homogenized with 8 mL of 0.2 M Hepes-NaOH buffer (pH 7.5, containing 5 mM MgCl_2_, 0.1% *β*-mercaptoethanol, 0.05% Triton X-100, 0.05% BSA, 2% crosslinking polyvinylpyrrolidone (PVPP), 1 mM EDTA, 1 mM EGTA, 10 mM AsA, 10 mM cysteine and 2% glycerol) and centrifuged at 10,000 × g for 20 min at 2°C. The SPS enzyme activity assay was carried out in a mixture containing 50 mM Hepes-NaOH buffer (pH 7.5), 15 mM MgCl_2_, 1 mM EDTA, 5 mM NaF, 16 mM UDPG, 4 mM fructose-6-phosphate (F-6-P), 20 mM glucose-6-phosphate (G-6-P) and enzyme extract. After incubation of this system for 30 min at 30°C, the reaction was terminated with 2 M NaOH at 100°C for 10 min. After cooling, 1 mL of 0.14% anthraquinone was added to the reaction system, and the system was kept at 40°C for 20 min. Then, the absorbance at 620 nm was recorded. The SSs enzyme activity measurement was carried out in a mixture containing 80 mM Hepes-NaOH buffer (pH 8.5), 5 mM DTT, 5 mM NaF, 15 mM UDPG, 100 mM fructose and enzyme extract. After incubation of this system for 30 min at 30°C, the reaction was terminated with 2 M NaOH at 100°C for 10 min. After cooling, 1 mL of 0.14% anthraquinone was added to the reaction system, and the system was kept at 40°C for 20 min, and the absorbance at 620 nm was recorded. For SSc enzyme activity measurement, the reaction mixture containing 80 mM Mes buffer (pH 8.5), 5 mM NaF, 5 mM UDP, 100 mM sucrose and enzyme extract was incubated at 30°C for 30 min. The reaction was terminated with 0.03 M DNS at 100°C for 10 min. After cooling, the absorbance at 540 nm was recorded. SSc activity is expressed as the amount of glucose produced every minute, whereas SPS and SSs activities are expressed as the amount of sucrose synthesized per minute (Zhou et al., 2018; Li et al., 2019).

### 2.8 Analysis of related genes

Plant total RNA extraction was conducted as follows: total RNA from citrus leaves and fruits was extracted and purified using a Spin Column Plant Total RNA Purification Kit (Sangon Biotech, China). The integrity of the RNA was evaluated by 1% agarose gel electrophoresis. An Aurora-800 ultramicro spectrophotometer (Aurora, HIPIE, China) was used for spectrophotometric analysis, and the concentration and purity of the extracted RNA were calculated from the A260/A280 ratio. RNA was transcribed into cDNA using a HiScript III 1st Strand cDNA Synthesis Kit with gDNA wiper (R323-01, Vazyme, China).

Detection of relevant genes was performed as follows: five antioxidant enzyme protein genes (*CsFe-SOD*, *CsMn-SOD*, *CsCu/Zn-SOD*, *CsPOD* and *CsCAT1*), three sucrose synthase-related genes (*CitSPS1*, *CitSPS2* and *SUS*) and two sucrose transporter-related genes (*SUC3* and *SUC4*) were selected from the NCBI database. The primer sequences are shown in **Supplementary Tables S2**, **S3**. The primers were synthesized by Wuhan Tianyi Huiyuan Biotechnology Co., Ltd., and then used for qRT‐PCR. cDNAs were reverse transcribed as templates using an ABI7500 real-time fluorescent quantitative PCR instrument (ABI7500, Applied Biosystems, United States) and a fluorescent dye (Taq Pro Universal SYBR qPCR Master Mix, Vazyme, China) by qRT‐PCR amplification. Three biological replicates were set up for each sample, and the internal reference gene was *Actin*. The 2^-ΔΔCt^ method was used to calculate the relative expression level of the genes (Livak and Schmittgen, 2001).

### 2.9 Statistical analysis

The results are expressed as the means ± standard errors (SE). SPSS Statistics 19.0 software (IBM Corporation, USA) was used for statistical analysis. Duncan’s test (*P* < 0.05) was used to evaluate the treatment effect. Origin 21.0 software (OriginLab Corp, USA) was used for image production and principal component analysis.

## 3 Results

### 3.1 Effect of AOS on citrus fruit quality

**Figure 2:**
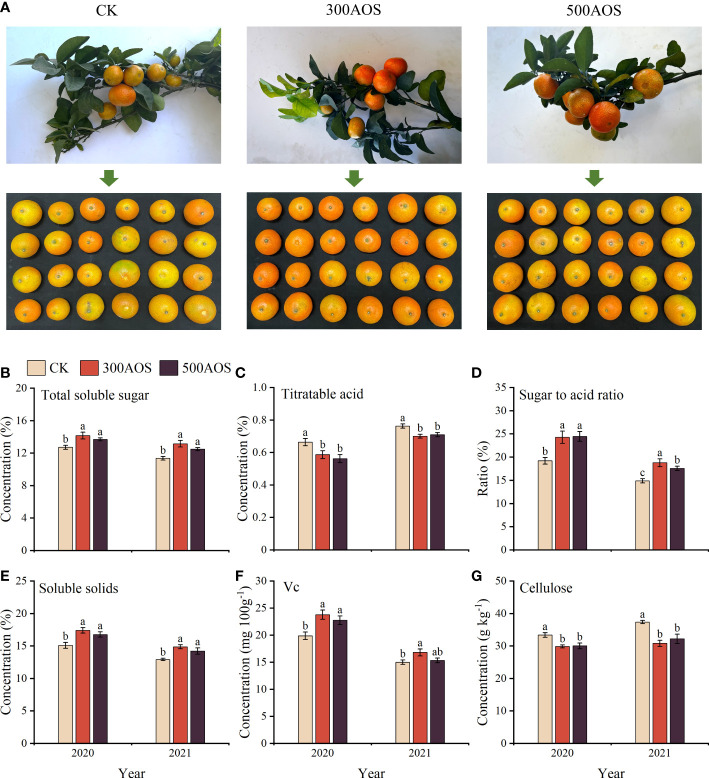
Effect of AOS on citrus fruit quality. Citrus fruit phenogram **(A)**, total soluble sugar content **(B)**, titratable acid content **(C)**, sugar to acid ratio **(D)**, soluble solids content **(E)**, Vc content **(F)** and cellulose content **(G)**. Different lowercase letters indicate significant differences among the treatments, based on the Duncan test (*p* < 0.05, *n* = 4).

As shown in **Figure 2**, AOS spray application significantly improved the quality of citrus fruits at harvest. Compared with the control, the soluble sugar content of citrus fruits increased by 7.74–11.36% and 9.88–15.79%, respectively (**Figure 2B**). The soluble solid content increased by 11.13–15.35% and 9.98–15.16%, and the fruit sugar-acid ratio was also significantly increased (**Figures 2D, E**). Compared to the CK treatment, the fruit Vc content in the 300AOS treatment increased significantly in both years, while the fruit Vc content in the 500AOS treatment increased significantly in 2020 but not in 2021 (**Figure 2F**). In addition, both the 300AOS and 500AOS treatments significantly reduced fruit titratable acid and flesh cellulose contents in both years of the experiment (**Figures 2C, G**). This result indicates that spray application of 300-500 mg L^-1^ AOS can effectively improve the nutritional and taste quality of citrus fruits by increasing sugar accumulation and decreasing the titratable acid and cellulose contents.

### 3.2 Effect of AOS on the antioxidant system of citrus leaves

**Figure 3:**
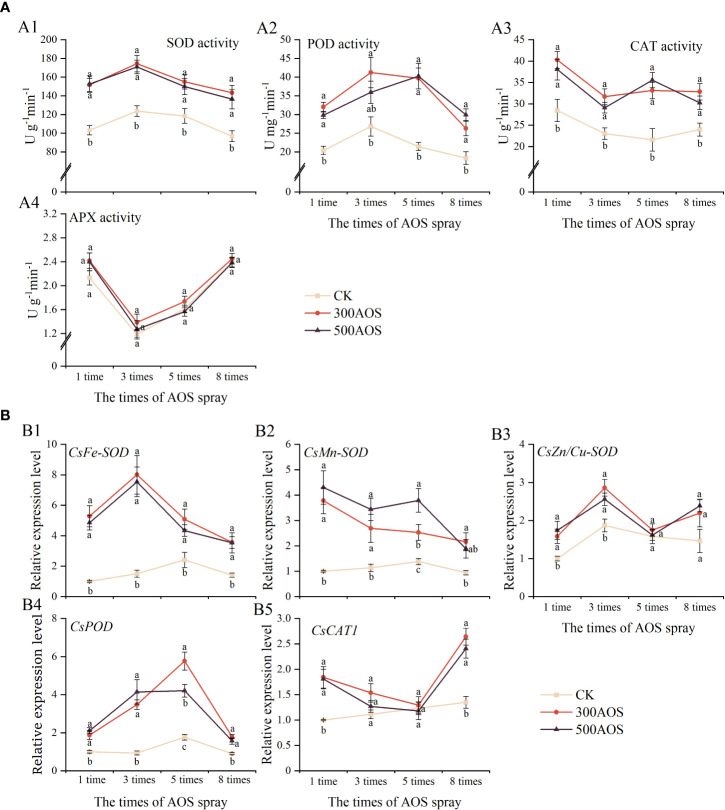
Effect of AOS treatment on the antioxidant system of citrus leaves. Antioxidant enzyme activity **(A)**, the expression of antioxidant enzyme-related genes **(B)**. Different lowercase letters indicate significant differences among the treatments, based on the Duncan test (*p* < 0.05, *n* = 4).

To investigate the effect of AOS spray application on the antioxidant system of citrus leaves, the activities of leaf antioxidant enzymes and the expression of related genes were measured. As shown in **Figure 3A**, AOS spraying significantly enhanced the activities of antioxidant enzymes (SOD, POD, and CAT) during citrus leaf development but had no significant effect on APX activity. Compared with the control, the leaf of SOD (30.86–47.91%), POD (43.12–84.95%) and CAT (36.83–53.55%), enzyme activities increased, respectively, throughout the treatment period for 300AOS treatment, while the leaf of SOD (26.63–47.85%), POD (33.94–87.70%) and CAT (25.96–64.81%) enzyme activities increased for 500AOS treatment. There was no significant difference in antioxidant enzyme activities between the two AOS levels (**Figures 3A1–A3**).

The expression of antioxidant enzyme-related genes in citrus leaves was measured, and the results are shown in **Figure 3B**. AOS spray application mainly affected the expression of the leaf *CsFe-SOD*, *CsMn-SOD* and *CsPOD* genes, while there was no significant effect on the expression of the *CsCu/Zn-SOD* and *CsCAT1* genes. Compared with the control leaves, the expression of *CsFe-SOD*, *CsMn-SOD* and *CsPOD* in 300AOS-treated leaves was upregulated 2.11–5.33, 1.83–3.78 and 1.90–3.71 fold, respectively, in the four sampling periods, and the expression of *CsFe-SOD*, *CsMn-SOD* and *CsPOD* in 500AOS-treated leaves was upregulated 1.80–5.02, 2.74–4.30 and 1.75–4.42 fold, respectively (**Figures 3B1, B2, B4**). However, the expression of the *CsCAT1* gene was significantly upregulated only after the 1st and 8th AOS spray cycles (**Figure 3B5**). The expression of these genes showed a consistent pattern with the changes in antioxidant enzyme activities, and the peak upregulation of leaf antioxidant enzyme-related gene expression also occurred mainly after the 3rd and 5th AOS treatments. This result indicates that AOS spray application can rapidly induce the activation of the leaf antioxidant system and increase antioxidant enzyme activity, which may help optimize the survival environment of the leaves.

### 3.3 Effect of AOS on photosynthesis in citrus leaves

**Figure 4:**
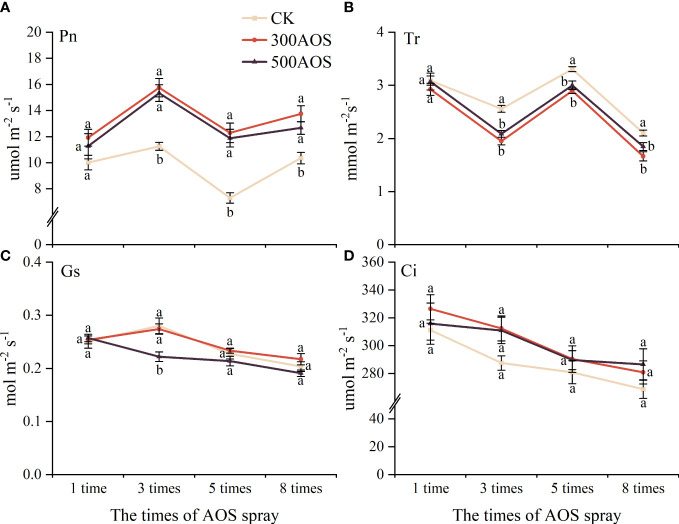
Effect of AOS treatment on photosynthesis in citrus leaves. Net photosynthetic rate **(A)**, transpiration **(B)**, stomatal conductance **(C)** and intercellular CO2 concentration **(D)**. Different lowercase letters indicate significant differences among the treatments, based on the Duncan test (*p* < 0.05, *n* = 4).

As shown in **Figure 4**, compared with the control, the Pn in citrus leaves with 300AOS and 500AOS did not change significantly after the 1st spraying but increased by 32.75–68.32% and 22.32–62.68% from the 3rd spraying, respectively. The Tr of leaves also did not change significantly after the 1st AOS spraying and decreased by 12.25–23.73% and 9.17–18.46%, respectively, from the 3rd AOS spraying (**Figures 4A, B**). In addition, AOS spray application significantly reduced the Gs of leaves only at 3rd spray. AOS spraying had no significant effect on leaf Ci (**Figure 4D**). The effect of AOS spray application on leaf photosynthesis was not immediate, and there was no significant difference between the two AOS concentrations, indicating that leaf photosynthesis did not increase with the increasing AOS concentration.

### 3.4 Effect of AOS on leaf assimilation products

Leaves are the main source of fruit sugars, and AOS spray application facilitated the accumulation of leaf assimilation products. As shown in **Table 1**, compared with the control, both the 300AOS and 500AOS treatments significantly increased the total chlorophyll and Chl a levels in citrus leaves at harvest, and 300AOS was also significantly higher than the 500AOS treatment. In addition, compared with the control, the leaf of soluble sugar (12.96%), protein (14.41%) and starch (10.98%) contents increased, respectively, throughout the treatment period for 300AOS treatment, while the leaf of soluble sugar (8.43%), protein (8.73%) and starch (9.33%) contents increased for 500AOS treatment, but there was no significant difference between the two AOS treatments (**Table 1**).

**Table 1:** Effect of AOS on assimilation products of citrus leaves.

Treatments	Total Chl (mg g^-1^)	Chl *a* (mg g^-1^)	Chl *b* (mg g^-1^)	Chl *a/b*	Soluble sugar (mg g^-1^)	Protein (mg g^-1^)	Starch (mg g^-1^)
CK	1.04 ± 0.01c	0.81 ± 0.02c	0.23 ± 0.02a	3.57 ± 0.32b	23.84 ± 0.64b	2.29 ± 0.08b	46.53 ± 1.22b
300AOS	1.38 ± 0.03a	1.13 ± 0.03a	0.25 ± 0.08a	5.09 ± 0.34a	26.93 ± 0.72a	2.62 ± 0.06a	51.64 ± 1.27a
500AOS	1.25 ± 0.01b	1.05 ± 0.03b	0.21 ± 0.05a	4.92 ± 0.58a	25.85 ± 0.55a	2.49 ± 0.04a	50.87 ± 2.20a

Letters behind the values in the same column indicate significant difference at different treatments, p < 0.05.

### 3.5 Effect of AOS on sugar metabolism in citrus fruits

**Figure 5:**
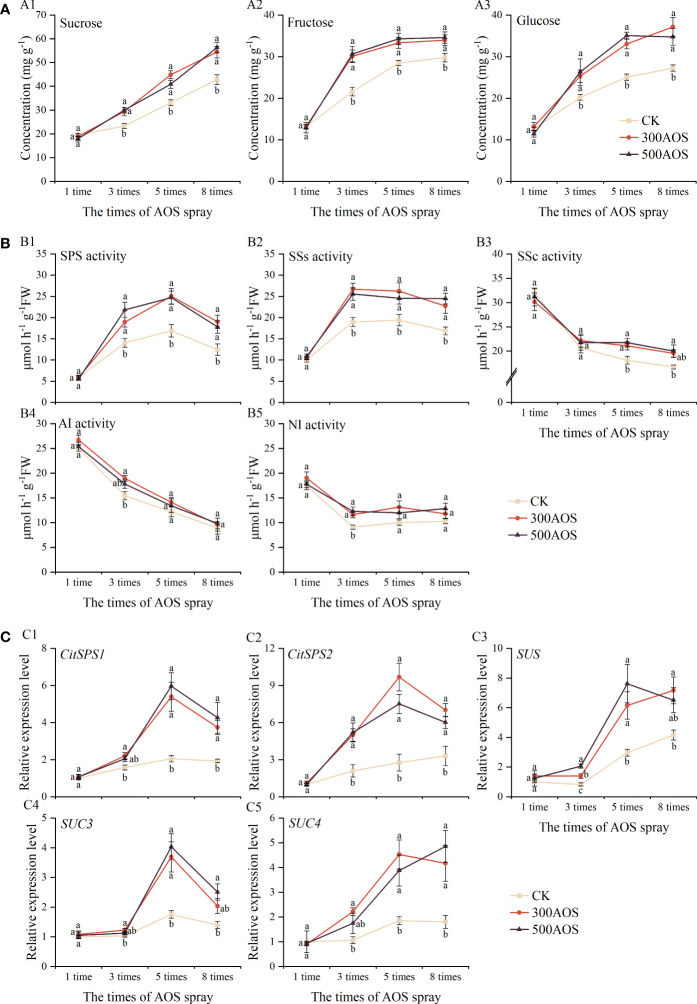
Effect of AOS treatment on sugar metabolism in citrus fruits. Sucrose, fructose, and glucose contents **(A)**, sucrose-related metabolic enzyme activities **(B)**, the expression of sucrose metabolism and transporter related genes **(C)**. Different lowercase letters indicate significant differences among the treatments, based on the Duncan test (*p* < 0.05, n = 4).

The main components of soluble sugars in citrus fruits are sucrose, fructose and glucose, of which sucrose is the main factor affecting citrus sweetness and is the main type of sugar accumulated in citrus fruits. As shown in **Figure 5A**, AOS spraying significantly increased the contents of sucrose, fructose and glucose in fruits. Compared with the control, the sucrose, fructose and glucose contents of both the 300AOS and 500AOS treatments were not significantly different after the 1st spraying. The sucrose content increased by 26.18–35.70% and 23.65–31.57%, the fructose content increased by 14.07–39.56% and 16.16–42.00%, and the glucose content increased by 25.26–36.23% and 27.44–39.97% in the 300AOS and 500AOS treatments, respectively, starting after the 3rd spraying. The incremental impact of AOS application on sucrose content was higher at the end of fruit development, while the incremental impact on fructose and glucose occurred mainly at the early stage of fruit development, which might be related to the pattern of sugar accumulation in citrus fruits themselves.

Sucrose–related metabolic enzyme activities were measured during the accumulation of sugar in fruits (**Figure 5B**). AOS application was found to significantly increase the activity of sucrose synthesis-related enzymes. Compared to the control, there were no significant differences in the activities of both SPS and SSs after the 1st spraying in the 300 AOS and 500 AOS treatments, but between the 3rd AOS spraying cycle and the 8th spraying cycle, the activities of SPS increased significantly by 34.94–53.29% and 43.34–55.46%, while the activities of SSs increased significantly by 34.61– 40.78% and 34.93–45.18%, respectively **(Figures 5B1, B2**). In addition, the activity of SSc in the 300AOS treatment increased significantly only from the 5th spraying cycle to the 8th spraying cycle, and the activities of AI and NI enzymes increased significantly only in the samples at the 3rd spraying cycle. While the activity of SSc in the 500AOS treatment also started to increase significantly from the 5th spraying cycle to the 8th spraying cycle, the activity of NI only increased significantly in the samples at the 3rd spraying cycle, while there was no significant effect on AI (**Figures 5B3–B5**). The pattern of the effect of AOS treatment on the activities of enzymes related to sucrose metabolism was consistent with the changes in sugar fractions.

The expression of sucrose synthase-related metabolic and transport genes was further examined in the fruit. As shown in **Figure 5C**, compared with the control, there were no significant differences in the expression of sucrose metabolism-related genes (*CitSPS1*, *CitSPS2* and *SUS*) in both the 300AOS and 500AOS treatments after the 1st spraying. The expression of the *CitSPS1*, *CitSPS2* and *SUS* genes was significantly upregulated starting after the 3rd spray cycle, and all of those genes exhibited the highest fold upregulation of expression after the 5th spray cycle (**Figures 5C1–C3**). Compared to the control, AOS treatment also significantly upregulated the expression of the sucrose transporter genes *SUC3* and *SUC4* after the 3rd spray cycle, and the expression of all sucrose transporter-related genes also peaked after the 5th spray cycle (**Figures 5C4, C5**).

### 3.6 Effect of AOS on sugar accumulation in citrus under reduced leaf conditions

**Figure 6:**
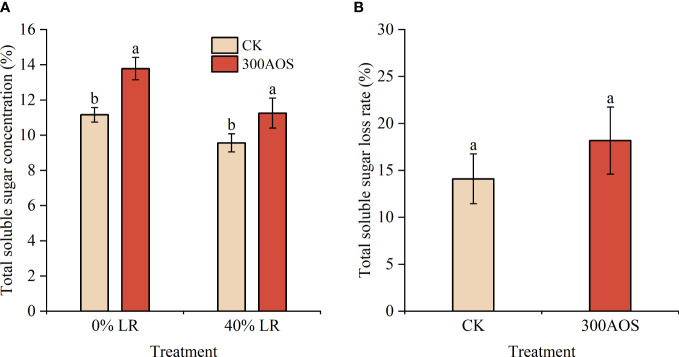
Effect of AOS on soluble sugars in citrus fruits under reduced leaf conditions. Total soluble sugar content **(A)**, Total soluble sugar loss rate **(B)**. Different lowercase letters indicate significant differences among the treatments, based on the Duncan test (*p* < 0.05, n = 4).

To determine whether there was a facilitative effect of AOS treatment on the transport of assimilated products from leaves to fruits, the content and rate of loss of soluble sugars in fruits under 40% leaf reduction were examined (**Figure 6**). Under both reduced and nonreduced leaf conditions, the fruit soluble sugar content was significantly higher in the 300AOS treatment than in the control treatment (**Figure 6A**). However, under reduced leaf conditions, the fruit soluble sugar loss rate was 14.1% for water spray application, which was lower than the fruit soluble sugar loss rate of 18.18% for the AOS treatment (**Figure 6B**). The leaves around the citrus fruit played a key role in soluble sugar accumulation in the fruit, and the loss of 40% leaves in the AOS treatment resulted in a higher rate in the soluble sugar loss, indicating that AOS treatment had a facilitative effect on the transport of assimilated products from the leaves.

### 3.7 Principal component analysis of AOS on citrus leaf and fruit-related indicators

**Figure 7:**
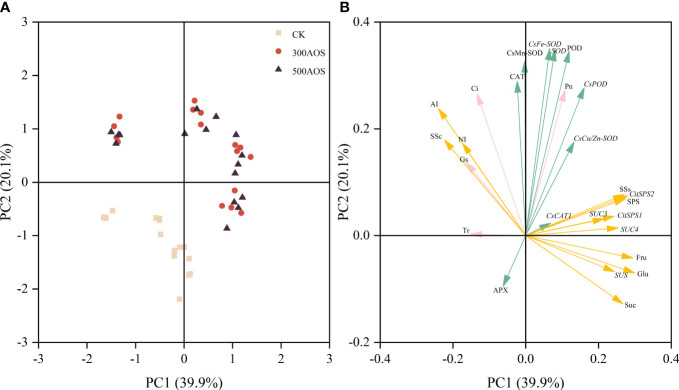
Principal component analysis of relevant indicators of leaf antioxidant system, photosynthesis and fruit sugar metabolism. The relationship between the scores of principal component analysis of related indicators and the amount of AOS applied **(A)**, principal component loading plot **(B)**.

All 26 leaf antioxidant, photosynthesis and fruit sugar metabolism-related index traits were grouped into two principal components (PC1 and PC2), explaining 60.0% of the total data variance. Most of the examined traits were distinguished by PC1, which was explained by a relatively large proportion of the variance (39.9%), while a lower proportion of the variance (20.1%) was indicated by PC2 (**Figure 7**). The concentration distribution showed that the AOS treatments and the control were almost completely separated from each other, indicating a relatively pronounced positive effect of AOS application on citrus indicator characteristics, but increasing the AOS concentration had little effect (**Figure 7A**). As shown in **Figure 7B**, the loaded indicators could be divided into three main categories, namely, the relationship between the leaf antioxidant system and photosynthesis, the relationship between fruit sugar accumulation and sugar metabolism, and the relationship between photosynthesis and fruit sugar metabolism. The relationship between both leaf antioxidant enzymes and related genes was at an acute angle to the net photosynthetic rate, the fruit soluble sugar fraction was at an acute angle to sucrose synthase and its related genes, and the net photosynthetic rate was also at an acute angle to fruit sucrose synthase (SPS, SSs) and fruit sucrose transport-related genes (**Figure 7B**). There was a significant positive correlation between photosynthesis and the leaf antioxidant system, and the accumulation of fruit sugars was mainly influenced by sucrose-related synthase and its gene expression, but photosynthesis also favored fruit sucrose transport and fruit sucrose synthase activity and related gene expression.

## 4 Discussion

### 4.1 Effect of AOS spray application on citrus fruit quality

AOS are important components of seaweed extracts with various biological activities and have great potential for application in agricultural production, especially in promoting crop growth, increasing yield, improving quality, and enhancing crop resistance (Zhang et al., 2020). For example, AOS can increase the levels of soluble sugars and soluble proteins in wheat leaves and improve the quality of wheat (Xu et al., 2003). Treatment of postharvest strawberries and kiwis with AOS maintained fruit hardness and soluble solid, organic acid, soluble sugar, and vitamin C contents (Bose et al., 2020; Liu et al., 2020). AOS spray application also increased the effective spike number and grain number of rice and increased rice yield (Zhang et al., 2014). However, relatively few studies have examined the effects of AOS application on citrus fruit quality. In the present study, the results of two years of field trials showed that AOS spray application (300 and 500 mg L^-1^) increased citrus fruit soluble solid content and Vc content, improved the soluble sugar and sugar-acid ratio, and reduced the levels of titratable acid and cellulose, thus improving the nutritional and flavor quality of citrus. This result may be related to the degree of aggregation of AOS. Iwasaki et al. demonstrated that AOS with DP 2-6 had the strongest promotion effect on root elongation in lettuce (Iwasaki and Matsubara, 2000). Zhang et al. (2013). found that AOS with DP 2-4 could induce root development in wheat. In contrast, the AOS prepared in this study had a DP range of 2-5 (**Supplementary Figure S1**), suggesting that these AOS may affect quality and sugar accumulation by promoting crop growth. In addition, some studies pointed out that AOS could enhance the uptake and utilization of elements by crops, which might help improve fruit quality. For example, increased levels of elements such as phosphorus (P) and potassium (K) promote fruit sugar and acid accumulation (Wu et al., 2021a; Wu et al., 2021b), and an increase in the levels of some elements could be essential for fruit photosynthesis, respiration, energy metabolism, and cell structure (Marschner, 2012).

### 4.2 Effect of AOS spray application on the antioxidant system and photosynthesis in citrus fruits

Soluble sugars are the main photosynthetic product of plants and the main form of carbohydrate metabolism and storage, and their accumulation in fruits is regulated by leaf photosynthesis and related enzymes. Jover et al. (2012). showed that high photosynthetic performance leads to high photosynthetic assimilate translocation from leaves to roots in New Holland orange, which facilitates the accumulation of sucrose and starch in the root system. Similar to plant growth regulators, previous studies have also confirmed that AOS can enhance photosynthesis and maintain crop growth in cucumber seedlings and cabbage under stress conditions (Qiao and Ouyang, 2013; Li et al., 2018). Liu et al. (2013). also suggested that AOS at different polymerization levels may promote root growth in wheat seedlings by stimulating photosynthesis, a process that also induces the expression of growth hormone-related enzymes and genes in the root system. In this study, no significant changes in leaf photosynthesis were observed after the 1st AOS spray cycle, while enhanced photosynthesis was detected after the third AOS spray cycle. AOS application significantly increased the Pn in leaves and decreased the Tr, which may be related to the fact that AOS activated the antioxidant system and improved the survival environment of leaves. After entering the plant, oligosaccharides can interact with cells as signaling molecules or act as inducers to regulate the growth pattern of plants and effectively regulate physiological activities related to the antioxidant system, photosynthesis and nutrient uptake, triggering the synthesis of different enzymes and activating various responses *via* changes in gene expression (Albersheim and Darvill, 1985; Ma et al., 2010). The antioxidant capacity of leaves is required not only to cope with oxidative damage caused by abiotic stress but also to maintain the photosynthetic capacity of plant leaves, and maintaining a high level of antioxidant capacity ensures that leaves maintain normal levels of carbohydrate production under altered environmental conditions (Liu et al., 2014). The intensity of photosynthesis in plants has a significant effect on the carbohydrate content in the plant body. Studies have also confirmed that photosynthesis affects the area of the leaf to varying degrees and has an impact on photosynthetic properties such as photosynthetic rate and carbon fixation (Fu et al., 2017; Overbeck et al., 2018). Leaf cell structural features also respond to the intensity of photosynthesis; for example, the thickness of fenestrations and spongy thin-walled tissues changes with light intensity (Ajmi et al., 2018). AOS have been shown to be effective in inducing antioxidant activity. The results of this study showed that the effect of AOS application on the leaf antioxidant system was more sensitive than that on photosynthesis. The antioxidant enzyme system of leaves was activated rapidly after the 1st AOS spray cycle, the activities of SOD, POD, and CAT were significantly increased, and the expression of genes related to antioxidant enzymes, such as *CsFe-SOD*, *CsMn-SOD*, and *CsPOD*, was significantly upregulated. This result may be related to the antioxidant capacity of AOS and the stress caused formed by the higher daily temperature in the orchard (**Supplementary Figure S2**). Available literature reports indicate that a class of biostimulants, such as luteolin and zeaxanthin, have a strong antioxidant capacity and can effectively inhibit the activity of oxygen radicals, which can effectively reduce damage to cellular structures caused by oxygen radicals during abiotic stresses (Jahns and Holzwarth, 2012). Falkeborg et al. (2014). also showed that enzymatically cleaved AOS can inhibit 100% of lipid oxidation and scavenge reactive oxygen species that cause damage. In this study, AOS spray application significantly increased the chlorophyll content of leaves, as well as the accumulation of carbohydrates such as soluble sugars and starch. Therefore, it can be inferred that AOS may facilitate the activation of the antioxidant system, optimize the survival environment of leaves, improve photosynthesis in the leaves, and thus promote the accumulation of assimilated products, providing a basis for the subsequent accumulation of sugars by the bank tissue, as similarly reported in the study of Antonietta et al. (Antonietta et al., 2017).

### 4.3 Effect of AOS spray application on sugar metabolism in citrus fruits

Sucrose, fructose and glucose are the major soluble sugar components in citrus, and the breakdown of sucrose plays an important role in regulating the accumulation of soluble sugars, which determines the sweetness of citrus fruits (Qiao et al., 2017). In citrus, sucrose is synthesized from the source leaves, transported through the bast and distributed to the depot tissue organs, and subsequently metabolized to fructose and glucose *via* AI and NI or to fructose and UDP-glucose in the fruits *via* SSc (Martinoia et al., 2012; Shen et al., 2017). This process leads to differences in sucrose concentrations between fruit and siliques, driving sucrose unloading in the tissues and thus promoting sucrose, fructose and glucose accumulation (Wind et al., 2010). The results of this study showed that AOS treatment significantly increased the levels of sucrose, fructose and glucose in fruits starting after the 3rd AOS spray cycle, which was related to the activity of enzymes and gene expression related to sugar metabolism. Katz et al. (2011). showed that sucrose accumulation in citrus fruits mainly originates from photosynthesis of leaves and fruit metabolism during the reproductive period, and this process is mainly influenced by sucrose-related metabolic enzymes (SSc, SSs, SPS) and translocases (AI, NI). Studies on Wenzhou honey tangerine showed that fruit sugar accumulation was consistent with SPS activity and was enhanced with the upregulation in the expression of two members of the SPS family, *CitSPS1* and *CitSPS2*. However, there was no significant relationship between sucrose storage and SPS activity in peach fruit (Komatsu, 2002; Vizzotto et al. al., 2010). Other genetic studies have also shown that sucrose-metabolizing enzymes play an important role in fruit sugar accumulation. For example, sucrose unloading capacity is reduced in young tomato fruit harboring an antisense sucrose synthase gene, while sucrose content is increased in melon fruits harboring an antisense acid convertase gene (Yu et al., 2008). In the present study, the enzyme activities of SPS and SSs related to sucrose synthesis showed a trend of increasing and then stabilizing throughout the reproductive period, while the enzyme activities of SSc, AI, and NI related to sucrose catabolism showed a gradual decrease, which was consistent with the results of previous studies (Wang et al., 2001). The measured enzyme activities and sucrose synthase gene expression levels were consistent with the change pattern of the sugar content. The activities of fruit sucrose synthases (SPS, SSs) were significantly increased starting after the 3rd AOS spray cycle, and the expression of the *CitSPS1*, *CitSPS2*, and *SUS* genes, related to sucrose synthases, was significantly upregulated. During fruit development, changes in sucrose metabolizing enzyme activities generally coincide with the accumulation of their transcriptional products (Braun et al., 2014). Thus, the promotion of sugar accumulation by AOS can likely be attributed to the enhancement of leaf photosynthesis during development and the correlation between AOS-triggered fruit sucrose synthase activity and activation of sugar metabolism-related gene expression, which was also confirmed in the principal component analysis (**Figure 7**). In addition, AOS treatment upregulated the expression of the sucrose transporter genes *SUC3* and *SUC4* (**Figure 5C**). The results of the leaf reduction experiments also showed that the reduction in fruit functional leaves in the AOS spray treatment increased the rate of fruit soluble sugar loss (**Figure 6**), indicating that AOS not only promoted the accumulation of leaf assimilation products but also played an active role in the transport of sucrose to the fruit, which is an interesting phenomenon that still needs to be further investigated.

## 5 Conclusion

In summary, 300-500 mg L^-1^ AOS application improved fruit quality and promoted sugar accumulation by regulating the following biological processes in citrus trees: (1) AOS application regulated the antioxidant system of citrus leaves. Specifically, the activities of antioxidant enzymes such as SOD, POD and CAT were increased, and the expression of antioxidant-related genes such as *CsFe-SOD*, *CsMn-SOD* and *CsPOD* was significantly upregulated, in leaves from the first AOS spraying. (2) The activation of the antioxidant system helped the leaves maintain good photosynthetic performance and improve the accumulation of assimilated products. Starting after the 3rd AOS spray cycle, the Pn of leaves increased significantly, and the content of leaf carbohydrates increased significantly at harvest. (3) AOS application promoted sugar metabolism and translocation in fruits. Starting from the 3rd AOS spray cycle, the sucrose, fructose and glucose contents were significantly increased, the activities of fruit sucrose synthases (SPS, SSs) were significantly increased, and the expression of sucrose synthase-related genes (*CitSPS1*, *CitSPS2*, *SUS*) and sucrose transporter-related genes (*SUC3*, *SUC4*) was significantly upregulated. Overall, this study found that AOS spray application may improve fruit sugar accumulation and quality by regulating the leaf antioxidant system, increasing the photosynthetic rate and assimilating product accumulation, and promoting sugar transfer from leaves to fruits. This study provides new insights into the quality improvement production of citrus fruits and the diversified utilization of marine oligosaccharide resources.

## Data availability statement

The original contributions presented in the study are included in the article/**Supplementary Material**. Further inquiries can be directed to the corresponding author.

## Author contributions

ZL: Performing - the experiments, Data curation, Writing - original draft, Writing - review and editing. SD: Performing - the experiments, Writing – reviewing and editing. BL: Performing - the experiments. CY: Performing - the experiments. HD: Writing-reviewing and editing. HS: Designing the study, Writing – reviewing and editing, Funding acquisition. All authors contributed to the article and approved the submitted version.
