# Supplementary material for: Spraying alginate oligosaccharide improves photosynthetic performance and sugar accumulation in citrus by regulating antioxidant system and related gene expression
Source: Front Plant Sci. 2023 Jan 30;13:1108848. doi: 10.3389/fpls.2022.1108848 (PMC9923110; doi:10.3389/fpls.2022.1108848)
Supplement: Supplementary file 1 [file DataSheet_1.docx]

***Supplementary Material***

**Spraying alginate oligosaccharide improves photosynthetic performance and sugar accumulation in citrus by regulating antioxidant system and related genes expression**

**Zhiming Li^1^, Songpo Duan^1^, Bosi Lu^1^, Chunmei Yang^1^, Hong Shen^1,2*^**

^1^ College of Natural Resources and Environment, South China Agricultural University, Guangzhou 510642, China

^2^ Guangdong Provincial Key Laboratory of Eco-Circular Agriculture, Guangzhou 510642, China

*** Correspondence author:**

Hong Shen

hshen@scau.edu.cn.

5 pages including 2 Figures and 3 Tables.


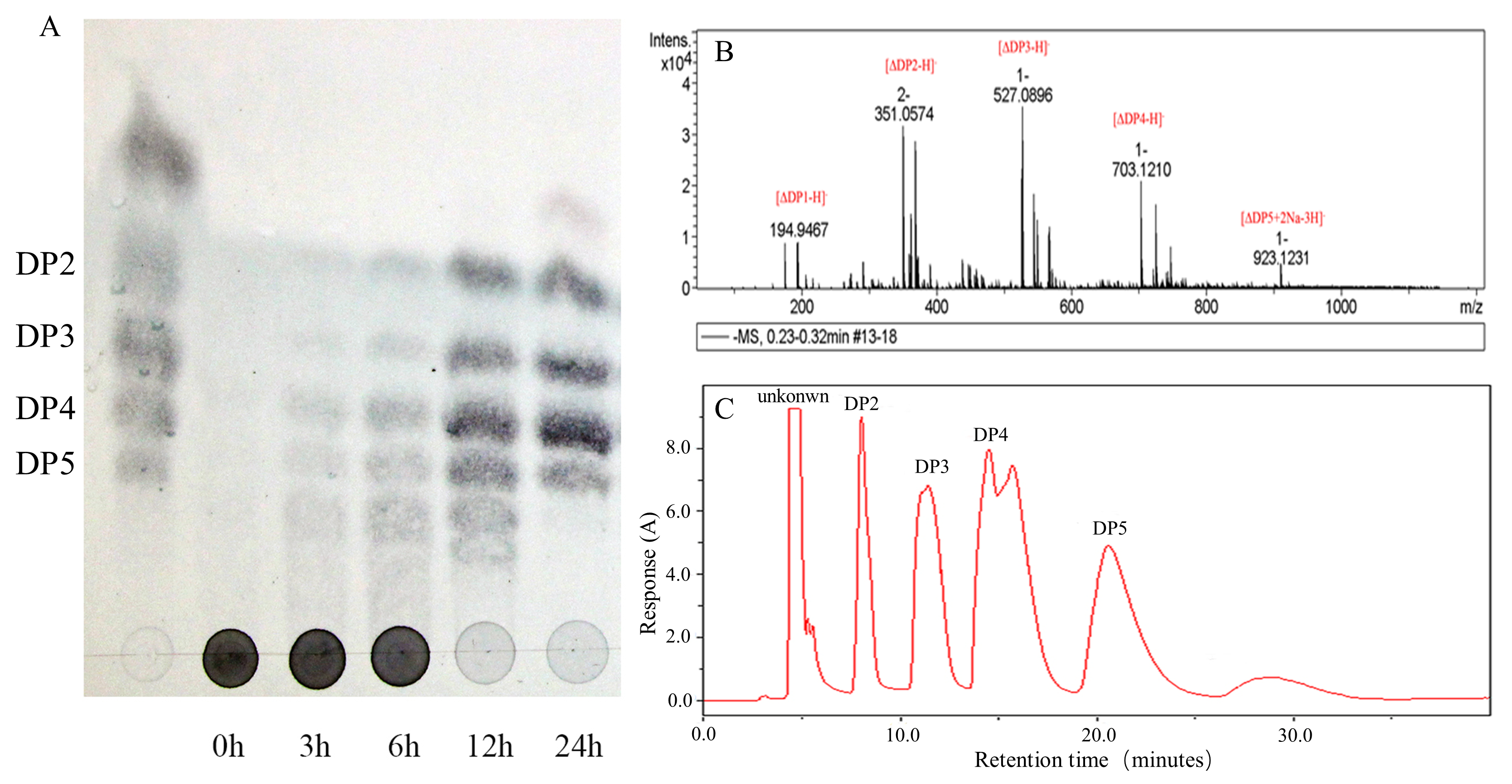
Supplementary Figure S1. Analysis of alginate sodium hydrolysates by TLC, ESI-MS and HPLC. TLC analysis (A), ESI-MS analysis (B) and HPLC analysis (C).
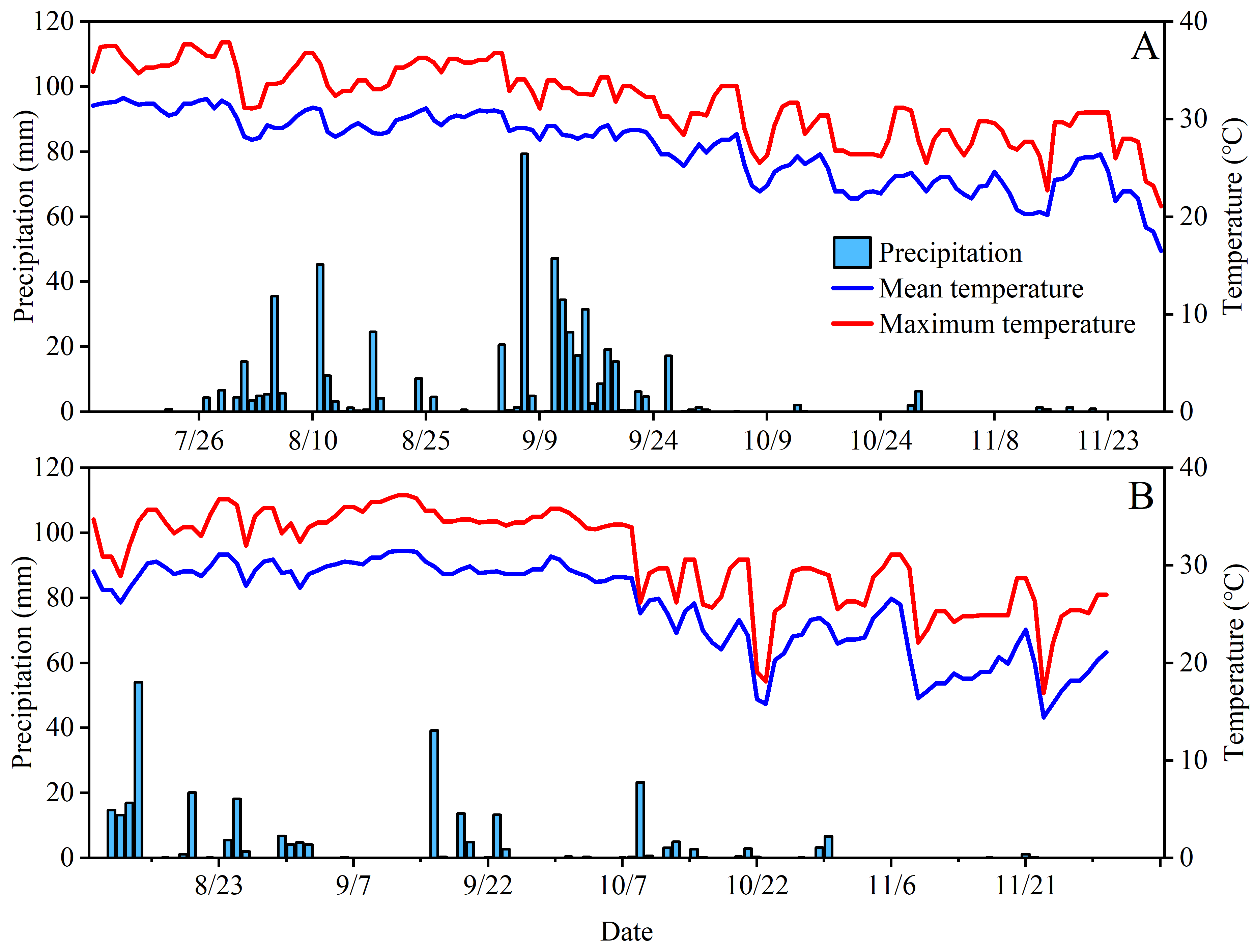
Supplementary Figure S2. Rainfall, maximum daily temperature and average daily temperature at the site during the citrus experiment. Year 2020 (A), Year 2021(B).

Supplementary Table S1. Citrus experimental plan for treatment and sampling time.

| Year 2020 | | |  | Year 2021 | | | |
| --- | --- | --- | --- | --- | --- | --- | --- |
| Date | Spraying time | Sampling time |  | Date | Spraying time | Sampling time | Sampling number |
| 2020-7-12 | **√** |  |  |  |  |  |  |
| 2020-7-26 | **√** |  |  |  |  |  |  |
| 2020-8-10 | **√** |  |  | 2021-8-9/10 | √ | √ | The 1st AOS spray cycle  (1 time) |
| 2020-8-25 | **√** |  |  | 2021-8-23 | √ |  |  |
| 2020-9-9 | **√** |  |  | 2021-9-7/8 | √ | √ | The 3rd AOS spray cycle  (3 times) |
| 2020-9-24 | **√** |  |  | 2021-9-22 | √ |  |  |
| 2020-10-9 | **√** |  |  | 2021-10-7/8 | √ | √ | The 5th AOS spray cycle  (5 times) |
| 2020-10-24 | **√** |  |  | 2021-10-22 | √ |  |  |
| 2020-11-8 | **√** |  |  | 2021-11-6 | √ |  |  |
| 2020-11-23 | **√** |  |  | 2021-11-21/22 | √ | √ | The 8th AOS spray cycle  (8 times) |
| 2020-12-10 |  | **√** |  | 2021-12-5 |  | √ |  |

Supplementary Table S2.Antioxidase related real-time fluorescent quantitative PCR primers.

| Gene | Primer sequences |
| --- | --- |
| *CsFe-SOD* | FW: AGTAAGGAGCGGCGAGTA |
|  | RV: GTGGCTAATGCGGTGAAT |
| *CsMn-SOD* | FW: GGCGAGCCACCACATAGT |
|  | RV: CACCCTCAGCATTCATCTTTT |
| *CsCu/Zn-SOD* | FW: GGACCAGCATGGACTACAAGACC |
|  | RV: GGATGCCGGTGGAAGTGTTACC |
| *CsPOD* | FW: GGCTCAACTTGTCCACCTC |
|  | RV: TATCGTCGCCCTGTCTG |
| *CsCAT1* | FW: TAACAGTGGAGGAGCGAACA |
|  | RV: GGAGCCAGTGCTAAGGGT |
| *β-Actin* | FW: CCGACCGTATGAGCAAGGAAA |
|  | RV: TTCCTGTGGACAATGGATGGA |

Supplementary Table S3. Sugar metabolism related real-time fluorescent quantitative PCR Primers

| Gene | Primer sequences |
| --- | --- |
| *CitSPS1* | FW: GCTCCCTCCTTATCCTTTCGT |
|  | RV: AGCAGCAACAAGACATCGAG |
| *CitSPS2* | FW: GTTGAACTTGCCCGAGCCTT |
|  | RV: ACATCTCGTTCGGTTCACCA |
| *CitSUS* | FW: AGAGTCACACTGCTTTCACTC |
|  | RV: GCTCATATCAGCACCAGGAG |
| *CitSUC3* | FW: AGCATCACCCGTCTCAAGTT |
|  | RV: TATGTTCGCCTGGTTCAAGA |
| *CitSUC4* | FW: CATGACCAATCTGCTCCTTT |
|  | RV: CATCCCAACCATGTTAGAGC |
| *β-Actin* | FW: CCAAGCAGCATGAAGATCAA |
|  | RV: ATCTGCTGGAAGGTGCTGAG |
